# A scrutiny of the relationship between cognitive load and difficulty estimates of language test items

**DOI:** 10.1186/s40468-022-00163-8

**Published:** 2022-06-15

**Authors:** Shadi Noroozi, Hossein Karami

**Affiliations:** Faculty of English Languages and Literatures, University of Tehran, Tehran, Iran

**Keywords:** Cognitive load theory, Perceived level of difficulty, Response time, Multiple-choice language items, Difficulty estimates, Rasch model

## Abstract

Recently, researchers have expressed their growing concern over the scrutiny of language test items in light of cognitive load theory (CLT). While cognitive load has been central to language learning research, it has not enjoyed due attention in high-stakes language tests. The current study set out to delve into the relationship between difficulty estimates and cognitive load of language test items. To measure cognitive load, examinees’ perceived level of difficulty and response time were considered. In this regard, empirical data were collected from 60 MA students and graduates through a quantitative correlational design. The current study further employed the Rasch model to estimate difficulties of the vocabulary and grammar items of the Iranian university entrance examination (IUEE) for MA in English majors held in 2018 and 2019. The study’s findings revealed statistically significant correlations between difficulty estimates and perceived level of difficulty for vocabulary items. As for grammar items, no statistically significant correlations were detected between the variables. Whereas the results indicated strong positive correlations between response time and difficulty estimates regarding vocabulary items, no statistically significant correlations were observed between the variables concerning grammar items. All in all, perceived level of difficulty, response time, and difficulty estimates appeared to be sound indicators of cognitive load with respect to vocabulary test items, but not with regard to grammar test items. The implications of the findings will be discussed.

## Introduction

In recent years, the investigation of language test items, using cognitive load theory (CLT), has garnered researchers’ attention (e.g., Dindar et al., 2015). As large-scale language tests have grave consequences for the stakeholders (Shohamy et al., 1996) and accomplishing any intellectual task can have impacts on examinees’ cognitive processes (Sweller, 1994), test items can also affect human cognitive processes. As the cognitive processes provoked by test items can play a crucial role in test takers’ performance, the analysis of the cognitive load of test items has become of paramount importance (Ehrich et al., 2021; Prisacari & Danielson, 2017). On the other hand, performance can reflect examinees’ cognitive processes (Gass et al., 2013). Hence, the cognitive load imposed by tasks should be commensurate with their difficulties.

The 1980s saw the emergence of a cognitive theory known as CLT proposed by Sweller. Theoretically, three categories of loads (i.e., intrinsic, extraneous, and germane) are underlying CLT. Intrinsic load is concerned with the innate nature and main characteristics of test items and has a close connection with task difficulty (Paas, Tuovinen, et al., 2003). Applied to various fields, this theory can explain the cognitive load related to instructional materials, teaching, learning (Sweller et al., 1998, 2019), and multimedia learning (Brünken et al., 2003).

Delving into the load of test items has also been a continuing concern within this framework. Thus, it is not entirely surprising that the exploration of test items in such fields as mathematics, chemistry, and algebra has gained momentum, with the hope of broadening the understanding of and improving item functioning by the close scrutiny of load patterns of items (Gvozdenko & Chambers, 2007; Prisacari & Danielson, 2017; Sweller et al., 2011).

In the examination of task complexity or difficulty through CLT, cognitive psychologists appear to have depended on subjective measures of perceived level of difficulty and perceived mental effort (e.g., Prisacari & Danielson, 2017), objective behavioral measures, including the secondary task technique and response time (e.g., Dindar et al., 2015; Ponce et al., 2020), or physiological measures consisting of electroencephalography (EEG) (e.g., Antonenko & Niederhauser, 2010). Researchers have employed several measures simultaneously to provide further insights into the cognitive load of tasks (e.g., Leppink, 2017).

However, some physiological (e.g., fMRI) and objective behavioral measures (e.g., the secondary task technique) seem to pose some problems, including being intrusive and needing special expertise (Ehrich et al., 2021) or imposing an extra load on the examinees’ cognitive architecture, respectively (Sweller et al., 2011). On the other hand, psychometricians seem to have relied on the prevalent use of measures such as item difficulty estimates through various item response models to explore item functioning (e.g., Karami, 2010).

To have a methods triangulation and assess whether difficulty estimates correspond to cognitive load measures and whether they can also be assumed as meaningful objective indicators of cognitive load, further research is needed. To obviate the problems created by implementing intrusive cognitive load measures and the secondary task technique, there is also a need to implement the objective measure of response time. In addition, there is a dearth of research on the cognitive load of language test items in the Iranian context, and ignoring the load of test items may have adverse effects on test takers’ minds and performance. Hence, this study aimed to examine whether item difficulty estimates computed from the Rasch Model correspond to examinees’ perceptions and whether they match the response time spent on each multiple-choice language item of the MA Iranian university entrance examination (IUEE) for English majors.

The current study first presents the general background of CLT. Cognitive load measures are then reviewed, and the pertinent empirical studies are expounded on. The explanation of the Rasch model’s theoretical underpinnings is also provided, and the relevant studies are explicated. The sections of method, results, discussion, limitations and future directions, and conclusion are subsequently presented.

## Literature review

### Cognitive load theory

Of the psychological theories that generally deal with the possible associations between a psychological construct or trait and an observable phenomenon, cognitive load theory (CLT) focuses on the effect of instruction. Stated more precisely, CLT is concerned with the “unobservable” phenomena (i.e., cognitive load) that learners experience whilst accomplishing various tasks. The constructs of *cognitive load* and *learning* are at the heart of CLT*.* Many researchers have studied cognitive load in such a wide range of disciplines as cognitive psychology and instructional design (Sweller, 2010). As a framework, CLT can account for the associations held between human cognitive architecture, learning, and instructional design (Sweller et al., 1998).

Opening a line of inquiry to account for the comparability of instructional designs regarding difficulty, CLT is built on two memory stores of short- and long-term memory referring to working and storage memory, respectively (Martin, 2014). Working memory is a place for conscious cognitive processing that can process a limited number of elements to compare, contrast, and organize information (Sweller et al., 1998). Its limitation may impede learning when it comes to processing and connecting several task elements at the same time. This limitation is due to the fact that working memory capacity (WMC) may be surpassed in such a case (Chandler & Sweller, 1991).

On the other hand, task requirements or demands influence the amount of provoked load on the working memory (Sweller et al., 1998). Hence, the overload and underload of WMC are of concern in designing instructional materials (Sweller et al., 2011) because working memory’s function and hence individuals’ performance can be affected in both cases (Johannsen, 1979; Young et al., 2015).

Efficient learning indeed hinges on whether cognitive architecture and instructional materials correspond to each other proportionally (Sweller et al., 2011). Besides learning, performance, which involves response time, rate of errors, and endorsed answers, is not independent of the over-or under-load imposed by tasks (Paas, Renkl, & Sweller, 2003). More precisely, examinees’ cognitive architecture and performance can be influenced by the imposed cognitive load of task requirements (Gvozdenko & Chambers, 2007).

By extension, this theory can also deal with the distribution of the cognitive capacities of test takers while taking a test (Sweller, 1988). Of the three categories of cognitive load (i.e., intrinsic, extraneous, and germane), intrinsic load, in particular, is expected to have interwoven relationships with the difficulty of the task/item itself (Paas, Tuovinen, et al., 2003). Intrinsic load is associated with the inherent attributes of test items (de Jong, 2010).

At the heart of intrinsic load is *element interactivity* which can be defined as the concurrent processing of several elements in the WMC to gain an understanding of or learn a task (Sweller, 2010). Element interactivity can be assumed to be the origin of intrinsic load (Sweller, 1994; Sweller et al., 2011). The amount of intrinsic load is specified by the interactivity of the elements of an item which is directly connected with task difficulty (Paas, Renkl, & Sweller, 2003). The term *difficulty* refers to the inherent nature of information and learners’ knowledge and may have various origins (Sweller, 2020; Sweller et al., 2011).

As for cognitive load measures, error rate was the only index of cognitive processes in the past. Median response time has also been applied to measure difficulty (Pelánek et al., 2022). Thanks to the theory development, the implementation of diverse subjective, objective, physiological measures of cognitive load has thrived. Cognitive load can be measured through such subjective measures as perceived mental effort and level of difficulty (Paas, 1992), time estimation (Baralt, 2013), NASA- task load index (Hart & Staveland, 1988), and Leppink’s cognitive load scale (Andersen & Makransky, 2021).

As for perceived level of difficulty, researchers (e.g., Ayres, 2006) have used this self-report rating scale to gauge mental effort, which is problematic because mental effort and task difficulty, though related, are two different constructs. Unlike mental effort that involves a process and is not limited to task characteristics, difficulty is merely associated with the task itself (van Gog & Paas, 2008).

Although some scholars have challenged the reliability and sensitivity of mental effort (e.g., de Jong, 2010; Moreno, 2010), it is still used as the sound index of cognitive load in a number of studies (e.g., Prisacari & Danielson, 2017). Similarly, perceived level of difficulty has shown to have sound reliability and validity (Prisacari & Danielson, 2017). The benefits of the subjective rating scale are its simplicity, cost and time efficiency, non-intrusiveness, high face validity, and minimal interference with the primary task (O’Donnell & Eggemeier, 1986; Scheiter et al., 2020).

Objective behavioral measures can also reveal cognitive load (Brünken et al., 2010), such as the secondary task technique, response time, time-on-task, and task complexity. Moreover, objective physiological measures include electroencephalography (EEG), heart rate, eye-tracking, functional magnetic resonance imaging (fMRI), and event-related brain potentials. Several cognitive load measures are suggested to be implemented concurrently to paint a clearer picture of cognitive load (Leppink, 2017; Skulmowski & Rey, 2017).

### Cognitive load studies

Regarding the relationship between task complexity and perceived level of difficulty, a considerable body of research indicated that the most complex tasks were perceived to be the most difficult (Ayres, 2006; Dindar et al., 2015; Jung, 2018; Lee, 2014; Lee, 2019; Révész et al., 2016; Robinson, 2001; Sasayama, 2016).

In a study, Ayres (2006) identified the alterations in element interactivity in solving different algebraic problems and employed the subjective measure of perceived difficulty. The results revealed that the more complex the task, the higher the element interactivity, and hence the higher the intrinsic load. He reasoned that as element interactivity dealt with the innate nature or characteristics of a task, it was assumed that problem complexity stemmed from element interactivity, which was in line with Sweller’s (2006) reasoning.

In another study, Jung (2018), focusing on the relationship between task complexity and perceived difficulty level, examined whether task complexity influenced individuals’ performance on the multiple-choice reading comprehension questions. The findings revealed that the participants experienced a greater amount of cognitive load in the case of a more complex task, with no influence on their test scores. In an investigation into the validity of perceived task complexity as a predictor of task performance in comparison with the physiological measure of heart rate, Minkley et al. (2021) reported a significant negative correlation between perceived task complexity and task performance. They showed that the subjective measure was a better predictor.

To further assess cognitive load fluctuations while performing a task, objective online measures seem to provide more helpful evidence than subjective ones (Paas & van Merriënboer, 1993). Due to a direct association between learners’ behaviors and learning processes, several behavioral measures of the secondary task technique, time-on-task, and task complexity all are viewed as the indicators of cognitive load (Brünken et al., 2010; Sweller et al., 2011; Sweller et al., 2019).

Numerous studies employed reaction time of the secondary task technique to gauge the load of different tasks with varying degrees of complexity (e.g., Lee, 2019; Sasayama, 2016).

Sasayama (2016) conducted research to gauge the cognitive complexity of several narrative tasks through the implementation of time estimation, perceived difficulty, and the secondary task technique. She observed that the highest level of difficulty and the longest recorded time were pertinent to the most complex task.

Also, Lee (2019) investigated whether alterations in task complexity indeed led to variations in cognitive load by applying the same measures used by Sasayama (2016) in addition to stress; however, the tasks were different. Similar findings were found: The most complex tasks were rated the most difficult and enjoyed the longest reaction time.

In one of the most recent studies, Greenberg and Zheng (2022) compared the tactile detection response task and the rhythmic tapping method to examine the interference of secondary tasks and their sensitivity to cognitive processing. They emphasized the interference caused by this technique and showed that it depended on the modality.

In contrast, several studies reported a lack of a relationship between reaction time and task complexity (e.g., DeLeeuw & Mayer, 2008; Révész et al., 2016). In a series of experiments, DeLeeuw and Mayer (2008) observed that reaction time did not appear to be sensitive to task complexity in their second experiment. Contrary to the results of their second experiment, those of their first experiment showed a correlation with the minimal effect size of .12.

To overcome the problems mentioned above related to the implementation of the secondary task technique (e.g., imposing extra load), another behavioral measure to assess cognitive load is response time employed in some studies (e.g., Dindar et al., 2015). A positive relationship between the amount of cognitive load and the invested time is expected according to Wright and Ayton (as cited in Lee, 2014). Marcus et al. (1996) carried out a series of experiments and concluded that there was a positive relationship between the number of element interactivity of a task and response time: the more complex the task, the longer the response time.

In another study, Gvozdenko and Chambers (2007) scrutinized each examinee’s response time on arithmetic test items to better understand their cognitive load. Their findings suggested that different questions displayed different cognitive processing with regard to response time, showing different levels of complexity. Further, Dindar et al. (2015), exploring the load of two types (static vs. graphic) of multiple-choice questions through the application of response time, accuracy rate, the secondary task technique, and perceived mental effort, reported a positive relationship between response time and task complexity. They showed that response time was a sound indicator of cognitive load.

The study designed by Ponce et al. (2020), focusing on response time and accuracy through the eye-tracking approach, aimed to evaluate the impact of an innovative interface (i.e., computerized mode) on the banked cloze tests. The role of response time in determining the cognitive load was highlighted: the longer the response time, the more complex the task, and the higher the cognitive load. Furthermore, Aryadoust et al. (2022) explored the impact of test methods on the listening performance and cognitive load of examinees through brain activity patterns, gaze behaviors, and listening performance. They observed that quicker eye movement was related to lighter cognitive load, and this lighter load was in turn associated with lower brain activity.

On the contrary, in a study by Pouw et al. (2016), no statistically significant correlations between response time and subjective cognitive load measures of mental effort and perceived difficulty were observed in an examination of the effect of two conditions (i.e., meaningful vs. non-meaningful) of physical engagement on various categories of competency. Their findings revealed that response time did not appear to be a sound cognitive load indicator. Still, other scholars (e.g., Lee, 2014) held that a small amount of response time showed a high level of cognitive load as learners refused to put effort into accomplishing the tasks when they became progressively difficult.

Although the abovementioned studies provided insightful findings into the application of various cognitive load measures, they ignored using another measure widely implemented in the realm of language testing. Difficulty estimates have recently shown to hold promise of assessing cognitive load (Ehrich et al., 2021). In this regard, estimating item difficulty through item response theory (IRT) and the Rasch model may reveal practical outcomes.

### The Rasch model

Item response theory (IRT), in general, and the Rasch model, in particular, have recently entered on the scene to contribute to cognitive load measures (Ehrich et al., 2021). The Rasch model (Rasch, 1960) is considered the least complicated and the most prevalent model of the large family of IRT models (Hambleton et al., 1991). In this model, the dichotomous item response is regarded as the observed dependent variable. On the other hand, trait level and item difficulty are considered independent unobservable variables. Hambleton et al. (1991) elaborated on the difficulty parameter *δ* of an item as the location parameter, which depends on person ability and can be expressed on the same continuum as that of person ability*.* The probability of endorsing an item is a function of the distance between the person located at *θ* and the item located at *δ*. The following formula indicates this:

$$p\ \left( xj=1\ |\ \theta, \delta j\right)=\frac{e^{\left(\theta -\delta j\right)}}{1+{e}^{\left(\theta -\delta j\right)}}$$

where *p* denotes the probability of endorsing an item *j* for an individual, *x* signifies the correct answer (*x* = 1), and *e* equals the constant value of 2.718. As an example, suppose the values of person ability and item difficulty are equal. In this case, a person has an equal chance either to endorse or to fail an item, which happens at the middle point of the item characteristics curve (ICC) where the rate of alterations changes (de Ayala, 2009; Embretson & Reise, 2000).

Items/curves differ only in location, and various locations represent different item difficulties. The curves converge but never cross. This implies that “the items maintain their difficulty order across score categories” (Embretson & Reise, 2000, p. 45). More difficult items are located at the higher end of the ability scale. Greater value of an item difficulty entails greater ability level to endorse it.

### Item response theory and cognitive processing: empirical studies

Now, turning our attention to the studies undertaken in the realm of assessment, we can observe that the complicated relationships between cognitive load measures (i.e., response time and perceived level of difficulty) and the task or item difficulty have also intrigued many researchers (e.g., Ehrich et al., 2021; Goldhammer et al., 2014; Prisacari & Danielson, 2017). Also, one of the latest studies highlighted the role of measures rooted in language testing to explain cognitive processes (Krell et al., 2021).

Goldhammer et al. (2014) set out to explore the relationship between response time and two types of tasks (i.e., the tasks of problem-solving and reading literacy) with varying degrees of difficulty. Concerning the problem-solving task that required higher-order thinking skills, there was a positive relationship between response time and task difficulty. By contrast, there was a negative relationship between task difficulty and response time in the reading task. Response time seemed to depend on and have an intricate relationship with task difficulty. It was reported that in some cases, the shorter the response time, the higher the probability of accomplishing a task correctly. In other cases, the larger the amount of time spent answering a task, the more probable it was to answer correctly. Generally, a longer response time seemed to be associated with a larger value of item difficulty, which is consonant with van der Linden (2009).

In another study, Prisacari and Danielson (2017), examining the cognitive load of chemistry tests in two modes (i.e., computer vs. paper) through item difficulty and perceived level of difficulty, demonstrated that there was a significant negative correlation between the variables, meaning easier test items were perceived to be less difficult. However, note that they made use of the item difficulty index computed from classical test theory.

On the other hand, there has been a growing trend towards applying the item difficulty computed by the Rasch model, and some scholars (e.g., Ehrich et al., 2021) have assumed it as a sound indicator of cognitive load. Ehrich et al. (2021), administering a Literacy and Numeracy (NAPLAN) test consisting of a numeracy test, a spelling subtest, and a grammar test, sought to explore whether item difficulty computed through the Rasch model was deemed a valid and reliable indicator of cognitive load and whether it measured cognitive load in line with perceived level of difficulty. They concluded that difficulty estimates were a sound indicator of capturing inherent task difficulty, that is, the more difficult the test item, the higher the perceived level of difficulty and the greater the amount of cognitive load.

A critical study by Krell et al. (2021) compared the model fit of the family of the Rasch models consisting of the one-parameter logistic model and the two explanatory psychometric models of linear logistic test models (i.e., baseline and extended). They analyzed the cognitive processes imposed by the item features (i.e., text readability and visual representations) and their influence on the overall difficulty of multiple-choice scientific reasoning instrument. The one-parameter logistic model showed a better fit; in addition, the extended linear logistic model demonstrated a better model fit compared with the baseline model.

All in all, contrary to the focus of cognitive load on test items in fields such as algebra, chemistry, arithmetic, scientific reasoning, and biology-related tasks, the cognitive load of language test items has not been closely investigated. The examination of reading literacy and problem solving (Goldhammer et al., 2014), cloze tests (Ponce et al., 2020), grammar items (Ehrich et al., 2021), language achievement tests (Dindar et al., 2015), and listening assessment (Aryadoust et al., 2022) has recently received attention. Nonetheless, the aforementioned studies were limited in a number of ways because they did not make use of cognitive subjective and objective load measures and difficulty estimates concurrently.

Also, there appears to be little consistency in the existing body of research with regard to the relationship between task difficulty and response time. On the one hand, there seems to be a consensus among several researchers on the positive relationship between task difficulty and response time (Aryadoust et al., 2022; Dindar et al., 2015; Gvozdenko & Chambers, 2007; Lee, 2019; Ponce et al., 2020). On the other hand, the results from several other studies showed no relationship between the variables (e.g., Pouw et al., 2016; Révész et al., 2014; Révész et al., 2016). Still, other studies underscored the complicated and non-uniform relationship between response time and task difficulty (e.g., Goldhammer et al., 2014). Hence, there appears to be controversy over scientific evidence in this respect.

Concerning the relationship between perceived difficulty and task complexity, almost every paper that has been written reported a positive relationship between the variables (Ayres, 2006; Jung, 2018; Lee, 2014; Lee, 2019; Prisacari & Danielson, 2017; Révész et al., 2016; Sasayama, 2016). However, it seems that there has been no detailed scrutiny of the large-scale multiple-choice language items using cognitive load measures and item difficulty.

The scarcity of research on the relationship between perceived difficulty and item difficulty of language test items and the observed inconsistencies in the relationship between item difficulty and response time necessitate empirical research to unite the realms of cognitive psychology and psychometrics. This union can be formed by making use of subjective and objective behavioral measures, as well as difficulty estimates. There are also some limitations to several studies mentioned earlier, such as being limited to the implementation of the family of the Rasch models to examine cognitive processes (Krell et al., 2021) or disregarding the subjective cognitive load measure of perceived difficulty (Aryadoust et al., 2022).

The concurrent use of cognitive load measures and item difficulty can provide insights into whether there is a correspondence between cognitive load measures and item difficulty. Indeed, the inspection of the relationship between item difficulty and perceived level of difficulty can unravel the (in)compatibility between the measures (Prisacari & Danielson, 2017). Moreover, despite the effect of item difficulty on the test takers’ performance concerning response time, there is a lack of research focusing on the association between response time and item difficulties in light of CLT.

It should be noted that Sweller et al. (2011) and Sasayama (2016) challenged the use of the secondary task technique due to confounding the load of the main or primary task with the additional load of the secondary task itself, and the interference was also underscored by Greenberg and Zheng (2022). Hence, to collect reliable and accurate data, the application of the objective measure (i.e., response time) not influenced by a secondary task has been suggested. Also, the simultaneous implementation of perceived level of difficulty and difficulty estimates computed from the Rasch model can better throw light on cognitive load patterns. It seems that no research has focused on the correspondence between cognitive load measures and difficulty estimates of the large-scale language tests in the Iranian context.

The current study sought to answer the questions below:

1. Is there any statistically significant correlation between the test takers’ perceived level of difficulty and the item difficulty estimates for each language test item?
2. Is there any statistically significant correlation between the item difficulty estimates and the response time for each language test item?

## Method

### Participants and context of the study

The participants attended the study in two phases. The first phase was necessary to compute item difficulty estimates because their calculation through the Rasch model required a large sample of at least 100 examinees, and it was not conceivable to have 100 participants due to the COVID-19 pandemic in Iran. Further, using the data collected under actual condition of examinations and taken from large samples of test takers would provide more dependable difficulty estimates. In the first phase, the participants (*N* = 2000), including 539 males and 1461 females, were randomly selected from a population who had sat the Iranian university entrance examination (IUEE) for an MA in English majors of Teaching English as a Foreign Language (TEFL), English Literature (EL), and Translation Studies (TS). Of the total population present at the exams in 2018 and 2019, 1000 examinees were screened from the examinees of each year such that out of the examinees sitting the examination in 2018, 751 test takers were female and 249 were male. Also, of the test takers who sat the exam in 2019, 710 examinees were female and 290 of them male.

In addition to the data from these examinees, further data were obtained from two sets of participants who attended the pilot and main studies in the second phase. The selection of both sets of participants was based on convenience sampling (Dornyei, 2007). All the participants were informed of the purpose of the study, and ethics approval was obtained from them. The first set of participants (*N* = 5) attended the pilot study to set the fixed timing of the presentation of multiple-choice questions on the screen. To record mental processing demands, the presentation time of stimuli is suggested to be fixed. Sixty MA students and graduates (35 female, 25 male) majoring in TEFL, EL, and TS, partook in the main study. They were aged between 21 and 39 (*M* = 27.28, SD = 4.41), studying at the University of Tehran, Allameh Tabataba’i University, and Alzahra University in Iran.

By administering the grammar part (100 items) of the OPT, the homogeneity of the participants’ level of language proficiency was also assured. Scores ranged from 75 to 94.5 such that the participants’ language proficiency levels were in the C1–C2 bands based on the common European framework of reference (CEFR). That is, they were advanced and very advanced users of the English language according to their obtained scores from the grammar part of the Oxford placement test (OPT) (Dave, 2004).

### Materials and instruments

The current study consisted of a proficiency test, multiple-choice vocabulary and grammar items of IUEE for MA in English majors, and a subjective self-report rating scale. After the administration of the OPT test, the participants who obtained the required levels of language proficiency were invited to attend the main study in which they were required to answer language test items of MA IUEE. This is because test takers’ right level of English proficiency is assumed to be revealed by their performance in the general English section of the examination. This section includes four parts: grammar (10 items), vocabulary (20 items), cloze passage (10 items), and reading comprehension (20 items). The current study focused on grammar (20 items) and vocabulary (40 items) multiple-choice questions of the two tests of IUEE for MA in English majors in a selected-response format administered in 2018 and 2019. The Grammar section included 20 items altogether such that half of the items belonged to the test held in 2018, and the other half was taken from the test held in 2019. There were also 40 vocabulary items in a way that half of the items were related to the test of 2018 and the other half to the test of 2019. No modifications to the original design and structure of the items were made because the purpose of the present study was to explore functioning of the same items of the entrance examinations. The researchers also checked the validity of the tests, and their validity was confirmed.

The present study also implemented the subjective self-report of perceived level of difficulty that is related to task difficulty per se. The rationale behind the prevalent use of self-reports is the simplicity of gleaning and analysis of subjective offline data (Paas, 1992; Paas & van Merriënboer, 1993). A 9-point Likert scale of *Level of Difficulty* was initially developed and validated by Bratfisch et al. (1972). The current study made use of the version of the 7-point Likert scale, ranging from 1 (*very easy*) to 7 (*very difficult*), validated by Prisacari and Danielson (2017). They also showed that this measure was an accurate and sensitive one to gauge the cognitive load of test items.

### Data collection procedure

Several steps were followed to collect the data. First, the dichotomous scores of examinees who had taken the examinations held in 2018 and 2019 were analyzed to calculate item difficulty estimates by the Winsteps software through the Rasch model (Linacre, 2013). The reason behind using the Rasch model rather than Item Response Theory (IRT) two- or three-parameter logistic models lies in the fact that “greater complexity does not automatically mean better” (Boone et al., 2014, p.455). Further, Boone et al. (2014) maintained that although more complex IRT models can consider such parameters as guessing and discrimination in addition to difficulty estimate, these models should be adjusted to fit the data, meaning these models depend on the data collected by a researcher. In contrast, the Rasch model is not modified to fit the data and hence is regarded as a definition of measurement.

Moreover, participants of the main study received instructions on the definition of perceived level of difficulty and took the test for about one hour. They had been instructed how to take the test and directed to answer language test items as correctly and as promptly as possible. Upon confirmation of their understanding of instructions, participants were asked to complete the language test items and the self-rating scale of perceived difficulty. Grammar and vocabulary items were presented sequentially in two blocks, each containing 20 and 40 items. Vocabulary and grammar blocks were counterbalanced across the participants to neutralize the effect of the order of presentation such that half of the participants answered the vocabulary items first and the other half first completed the grammar items. The order of test items in each block was also randomized by the Psychopy software. Before the presentation of each item, a centrally positioned fixation cross (i.e., 500 ms) was also displayed. The participants’ response time spent on each language item and their responses were recorded by the Psychopy software (Peirce et al., 2019) that records dependable response time spent on each test item with the exactness of millisecond. When each multiple-choice item was answered or completed, participants were directed to answer the 7-point Likert-type item about the perceived difficulty level of the item on a separate page on the screen. This procedure continued up to the end of the test.

### Data analysis

Difficulty estimates were computed through the Rasch model via Winsteps. To this end, the model-data fit, unidimensionality, and local independence were examined. In addressing the first research question, both Pearson product-moment and Spearman rho correlations were run due to the fact that Likert-type items or ranked data may sometimes be considered ordinal in nature (Boone et al., 2014; Pallant, 2016). Note that as the results were similar, only the results obtained from Pearson correlations are reported. To answer the second research question, Pearson’s product-moment correlation was run.

The normality assumption for response time and perceived difficulty level was checked by Shapiro–Wilk’s test (*p* > 0.05), and hence it was shown that this assumption was not violated. It should be noted that response times of only correct responses were taken into consideration because they seem to indicate cognitive load rather than those of incorrect answers. This approach to the analysis of time was also adopted and endorsed in several cognitive load studies (e.g., Lee, 2019; Révész et al., 2016; Sasayama, 2016). Also, note that the outcomes of grammar and vocabulary items are displayed in two separate sets: the first set showing the items pertinent to the test administered in 2019 and the second set representing the items related to the test held in 2018. This is because the presence of a second set of data can potentially assist in cross-validation of the results.

## Results

The first research question explored the relationship between the test takers’ perceived level of difficulty and difficulty estimates. As shown in Table 1, no statistically significant correlation was observed between the aforesaid variables. That is, the increase or decrease in the difficulty estimates of grammar items seems to have no association with the increase or decrease in their respective perceived level of difficulty. Although a non-significant relationship was found between difficulty estimates and perceived level of difficulty of the first set of grammar items, a small effect size of 0.16 was observed. The results indicate that the subjective cognitive load measure and difficulty estimates may not correspond in the delineation of difficulty or cognitive load of grammar items. Also, there is a significant correlation between perceived level of difficulty across sets (*r* = .583, *p* < .001).

**Table 1:** Pearson’s product moment correlation of difficulty estimates and perceived difficulty level (grammar items)

		1	2	3	4
1.1. Difficulty estimates grammar items (first set)	Pearson	–	.166	.046	.145
	Sig. (2-tailed)		.206	.725	.269
2. Perceiveddifficulty levelgrammar items (first set)	Pearson		–	.177	.583^**^
	Sig. (2-tailed)			.177	.000
3. Difficulty estimates grammar items (second set)	Pearson			–	− .094
	Sig. (2-tailed)				.475
4. Perceived difficulty level grammar items (second set)	Pearson				–
	Sig. (2-tailed)				

Table 2 shows the correlations between difficulty estimates and perceived level of difficulty with regard to the vocabulary section. It is apparent that difficulty estimates were significantly correlated with perceived level of difficulty of the first set of vocabulary items (*r* = .366, *p* < .001) with a medium effect size. There was also a significant positive correlation between difficulty estimates and perceived level of difficulty of the second set of vocabulary items (*r* = .693, *p* < .001). The results suggest that difficulty estimates can be assumed to indicate cognitive load in a similar way to perceived level of difficulty.

**Table 2:** Pearson’s product moment correlation of difficulty estimates and perceived difficulty level (vocabulary items)

		1	2	3	4
1. Difficulty estimate vocabulary items (first set)	Pearson	–	.366^**^	.485^**^	.383^**^
	Sig. (2-tailed)		.004	.000	.003
2. Perceived difficulty level vocabulary items (first set)	Pearson		–	.444^**^	.737^**^
	Sig. (2-tailed)			.000	.000
3. Difficulty estimate vocabulary items (second set)	Pearson			–	.693^**^
	Sig. (2-tailed)				.000
4. Perceived difficulty level vocabulary items (second set)	Pearson				–
	Sig. (2-tailed)				

The results of the correlational analyses of response time and difficulty estimates can be seen in Table 3. Obviously, no statistically significant correlation is observed between the variables, meaning that response time and difficulty estimates seem not to measure cognitive load in a similar way.

**Table 3:** Pearson’s product moment correlation of response time and difficulty estimates (grammar items)

		1	2	3	4
1. Response time grammar items (first set)	Pearson	–	.085	.640^**^	.136
	Sig. (2-tailed)		.520	.000	.301
2. Difficulty estimates grammar items (first set)	Pearson		–	.124	.046
	Sig. (2-tailed)			.345	.725
3. Response time grammar items (second set)	Pearson			–	− .151
	Sig. (2-tailed)				.249
4. Difficulty estimates grammar items (second set)	Pearson				–
	Sig. (2-tailed)				

As evident from Table 4, there was a significant positive correlation between response time and difficulty estimates of the first set of vocabulary items (*r* = .323, *p* < 0.05) with a medium effect size. On the other hand, the table illustrates a strong positive correlation between the variables concerning the second set of vocabulary items (*r* = .709, *p* < .001). Therefore, response time and difficulty estimates appear to measure cognitive load similarly.

**Table 4:** Pearson’s product moment correlation of response time and difficulty estimates (vocabulary items)

		1	2	3	4
1. Difficulty estimates vocabulary items (first set)	Pearson	_	.323^*^	.485^**^	.441^**^
	Sig. (2-tailed)		.012	.000	.000
2. Response time vocabulary items (first set)	Pearson		_	.445^**^	.780^**^
	Sig. (2-tailed)			.000	.000
3. Difficulty estimates vocabulary items (second set)	Pearson			_	.709^**^
	Sig. (2-tailed)				.000
4. Response time vocabulary items (second set)	Pearson				_
	Sig. (2-tailed)				

## Discussion

The current study attempted to ascertain whether the difficulty estimates obtained from the Rasch model match perceived level of difficulty and response time, and whether they can be regarded as indicators of cognitive load. The first research question aimed to examine the association between difficulty estimates and perceived level of difficulty. The results revealed no significant correlations between the aforementioned variables regarding the grammar items and significant correlations between the variables as for the vocabulary items.

It was shown that item difficulty estimates seemed not to correspond to perceived level of difficulty, suggesting that item difficulty may not be a sound indicator of or sensitive to cognitive load of the grammar items. Stated another way, cognitive load of the grammar items could not be captured by difficulty estimates in much the same way as perceived level of difficulty: The grammar items that the test takers perceived to be more difficult were not in fact more difficult. These findings seem to be in disagreement with the results of previous studies that highlighted a positive relationship between task complexity and perceived level of difficulty (Ayres, 2006; Dindar et al., 2015; Jung, 2018; Lee, 2019; Révész et al., 2016; Sasayama, 2016).

There can be several explanations for the lack of relationships between perceived level of difficulty and difficulty estimates of the grammar items. A conceivable reason may be that the test takers might have under- or over-estimated the item difficulty, an inevitable side effect of using self-reports (Ary et al., 2019). Another plausible explanation may be that the test takers’ perceptions of difficulty can vary as the type of task or item at hand changes (Lee, 2019). That is, there may be some inherent difference between vocabulary and grammar items.

As to the vocabulary items, the results seem to be in accord with those of previous studies, meaning that the more complex the items were perceived, the more difficult the items were in reality (Ayres, 2006; Ehrich et al., 2021; Jung, 2018; Lee, 2019; Prisacari & Danielson, 2017; Révész et al., 2016; Sasayama, 2016). Our findings mirror those of the study conducted by Prisacari and Danielson (2017) who reported a significant negative correlation between item difficulty and perceived level of difficulty and concluded that test items seemed to be perceived as less difficult when they became incrementally easy.

Further, the results of the present study are consonant with those of a recent study conducted by Ehrich et al. (2021) who concluded that difficulty estimates computed through the Rasch model were considered valid measures of cognitive load. Likewise, the individuals’ perceptions about difficulty of the vocabulary items were in line with their estimated item difficulties in our study. It was shown that participants’ judgements about difficulty in a general sense seemed to be congruent with the difficulties estimated by the Rasch model. However, this did not hold in the case of grammar items.

Notwithstanding the unexpected results of the grammar section, significant correlations between difficulty estimates and perceived difficulty concerning the vocabulary section were suggestive of the sensitivity of difficulty estimates to cognitive load. Together, these results indicate to jibe partially with the predictions. Our outcomes confirm that difficulty estimates appear to be meaningful indicators of the cognitive load of vocabulary items. They can indeed assume a dual role for difficulty estimates. Not only can they reveal information about the performance of test takers based on their correct answers to test items, but they can also be considered an index of cognitive load revealing evidence about the cognitive processing of examinees.

The second research question investigated the relationship between response time and difficulty estimates. To this end, Pearson correlations were run, and the results were mixed with reference to grammar and vocabulary sections, similar to those obtained in response to our first research question. Contrary to the expectations, the current study found no statistically significant correlations between the variables in grammar items. However, significant correlations between the aforesaid variables were observed in the vocabulary section.

Concerning the grammar section, our findings seem to be consistent with those of the previous studies that reported no relationships between response time and task difficulty (DeLeeuw & Mayer, 2008; Pouw et al., 2016; Révész et al., 2014; Révész et al., 2016). Our results revealed that response time, not sensitive to task complexity, failed to explain cognitive load of the grammar items. By contrast, our findings conflict with those of the studies that suggested a positive relationship between task complexity and response time (e.g., DeLeeuw & Mayer, 2008; Dindar et al., 2015; Ponce et al., 2020; van der Linden, 2009).

There are several explanations for finding no patterns in examining the relationship between response time and difficulty estimates in this regard. A possible explanation for these results can be different effects of task types on individuals’ perceptions (Lee, 2019). Another plausible explanation for this finding might be that the number of characters that grammar items contained was inequivalent and relatively large. This means a longer response time may indicate the time spent reading a large number of item characters rather than the time needed to reflect on finding the answer that can reveal the load or difficulty of an item. That is, a larger amount of time can not necessarily provide evidence about cognitive load.

Regarding the vocabulary section, the outcomes align with those of the previous studies (Aryadoust et al., 2022; Dindar et al., 2015; Gvozdenko & Chambers, 2007). It was underscored that when the task at hand became more demanding or more difficult, a longer time was needed to complete it, showing heavier cognitive load or cognitive processes. Response time and item difficulty seem to detect cognitive load of the vocabulary items similarly. The outcomes of the present study are also in partial agreement with those of the study carried out by Goldhammer et al. (2014) who emphasized the role of difficulty in getting an item correct: the more difficult the task, the longer the response time. The reason for the partial agreement is that Goldhammer et al. (2014) employed different types of tasks (i.e., reading literacy vs. problem-solving) and observed no uniform patterns of relationships. Hence, the type of task appears to be influential in determining its difficulty.

## Limitations and future directions

The current study shed light on whether difficulty estimates assess cognitive load of the language test items similar to the cognitive load measures. Moreover, our study highlighted the role of dependable response time in gauging load of the language test items. The findings in this study are subject to several limitations. One limitation is focusing solely on the vocabulary and grammar sections and discarding reading comprehension and cloze test parts on account of problems related to running the experiments on the Psychopy software.

The results may not also apply to the wider population of students because only MA English major students and graduates with advanced and very advanced levels of language proficiency attended the study. Hence, low proficiency groups of students and graduates were not considered. Further research is required to compare and contrast the performance and perception of low with high proficiency level groups because it has been reported that those with a high level of proficiency are more capable of differentiating the nuances of task complexity (Sasayama, 2016).

To better understand and clarify the criteria that the participants used to rate their perceived level of difficulty, retrospective interviews, and think-aloud techniques can provide insightful findings. Future studies should also be carried out to assess and compare the cognitive load of the least- to the most- complex test items. The categorization of test items and exploring their results may be interesting. An extraneous variable that may obscure the results is the lack of equivalent characters in the grammar section; thus, it is strongly recommended to use test items containing roughly the same number of characters in future investigations.

Ultimately, it is highly recommended that further studies compute item difficulty estimates in light of more complex item response theory (IRT) models (e.g., the three-parameter logistic model) to take discrimination and guessing parameters into account and hence minimize the effect of guessing, which is of concern in high-stakes exams (Hambleton et al., 1991). In addition, to examine the precision with which the difficulty estimates were computed, comparing the difficulty estimates obtained from different IRT models is recommended (Embretson & Reise, 2000).

## Conclusion

To our knowledge, this study is considered the first attempt to examine high-stakes language test items in light of cognitive load theory (CLT) using difficulty estimates in the Iranian context. The study delved deeply into whether difficulty estimates behave in the same way as the subjective cognitive load measure in reflecting load of language test items, and whether response time and difficulty estimates, as two objective measures, can detect cognitive load of test items in a similar way.

Our findings, overall, revealed that the predicted correlations were born out only for vocabulary items. One significant outcome was the congruence between perceived level of difficulty and difficulty estimates of vocabulary items. In other words, all the vocabulary items indicated to line up according to their difficulty as predicted; however, the grammar items turned out to behave with no specific pattern. Another major result was that difficulty estimates were deemed to capture cognitive load in line with response time; nevertheless, the response time recorded for the grammar items showed not to go together with their difficulty estimates. All in all, item difficulty estimate and response time revealed to be the sound indicators of cognitive load for vocabulary items.

Also, the findings of the current study can provide significant theoretical implications in connection with CLT. Applying item difficulty estimates and cognitive load measures can enhance researchers’ understanding of the item evaluation and the influence of language items’ loads on test takers’ minds. Besides, due to some criticism leveled against the use of subjective measures such as being subject to under- or over-estimation of individuals, the implementation of response time can contribute to CLT through providing helpful information about item difficulty, cognitive processes, and item functioning (de Jong, 2010; Ponce et al., 2020; van der Linden, 2009). Hence, the use of difficulty estimate and response time can assist in explaining the findings of cognitive load measures.

Moreover, these outcomes have implications within the field of language testing. Cognitive load theory can play a key role in the exploration of item functioning in psychometrics through the concurrent use of cognitive load measures. In this way, test designers can have a thorough grasp of the load and the function of the items they develop. This awareness can help test designers to proceed with caution in the test design, considering the possible detrimental effects of item malfunctioning on the test takers’ minds, such as overloading human cognitive resources or influencing test scores detrimentally In other words, test developers can examine whether the test items they design correspond to the characteristics being experienced or perceived by the test takers regarding difficulty and speed of processing. This can also minimize the danger of over-reliance on difficulty estimates as the sole statistical index of difficulty in item analysis.

## Data Availability

The data are available upon request from the authors.

## Abbreviations

CLT: Cognitive load theory
IRT: Item response theory
IUEE: Iranian university entrance examination
ICC: Item characteristics curve
OPT: Oxford placement test
CEFR: Common European framework of reference

## References

[CR1] Andersen MS, Makransky G (2021). The validation and further development of a multidimensional cognitive load scale for virtual environments. Journal of Computer Assisted Learning.

[CR2] Antonenko PD, Niederhauser DS (2010). The influence of leads on cognitive load and learning in a hypertext environment. Computers in Human Behavior.

[CR3] Ary D, Jacobs LC, Irvine S, Walker D (2019). *Introduction to research in education*.

[CR4] Aryadoust V, Foo S, Ng LY (2022). What can gaze behaviors, neuroimaging data, and test scores tell us about test method effects and cognitive load in listening assessments?. Language Testing.

[CR5] Ayres P (2006). Using subjective measures to detect variations of intrinsic cognitive load within problems. Learning and Instruction.

[CR6] Baralt M (2013). The impact of cognitive complexity on feedback efficacy during online versus face-to-face interactive tasks. Studies in Second Language Acquisition.

[CR7] Boone WJ, Staver JR, Yale MS (2014). *Rasch analysis in the human sciences*.

[CR8] Bratfisch O, Borg G, Dornic S (1972). *Perceived item-difficulty in three tests of intellectual performance capacity* (Report No. 29).

[CR9] Brünken R, Plass JL, Leutner D (2003). Direct measurement of cognitive load in multimedia learning. Educational Psychologist.

[CR10] Brünken R, Seufert T, Paas F, Plass J, Moreno R, Brünken R (2010). Measuring cognitive load. *Cognitive Load Theory* (pp. 181-202).

[CR11] Chandler P, Sweller J (1991). Cognitive load theory and the format of instruction. Cognition and Instruction.

[CR12] Dave A (2004). *Oxford placement test*.

[CR13] de Ayala RJ (2009). *The theory and practice of item response theory*.

[CR14] de Jong T (2010). Cognitive load theory, educational research, and instructional design: Some food for thought. Instructional Science.

[CR15] DeLeeuw KE, Mayer RE (2008). A comparison of three measures of cognitive load: Evidence for separable measures of intrinsic, extraneous, and germane load. Journal of Educational Psychology.

[CR16] Dindar M, Yurdakul IK, Dönmez FI (2015). Measuring cognitive load in test items: Static graphics versus animated graphics. Journal of Computer Assisted Learning.

[CR17] Dornyei Z (2007). *Research methods in applied linguistics*.

[CR18] Ehrich JF, Fitzgerald J, Howard SJ, Bokosmaty S, Woodcock S (2021). An item response modeling approach to cognitive load measurement. Frontiers in Education.

[CR19] Embretson SE, Reise SP (2000). *Item response theory for psychologists*.

[CR20] Gass SM, Behney J, Plonsky L (2013). *Second language acquisition: An introductory course*.

[CR21] Goldhammer F, Naumann J, Stelter A, Tóth K, Rölke H, Klieme E (2014). The time on task effect in reading and problem solving is moderated by task difficulty and skill: Insights from a computer-based large-scale assessment. Journal of Educational Psychology.

[CR22] Greenberg, K., & Zheng, R. (2022). Cognitive load theory and its measurement: a study of secondary tasks in relation to working memory. *Journal of Cognitive Psychology*, 1–19. 10.1080/20445911.2022.2026052.

[CR23] Gvozdenko E, Chambers D (2007). Beyond test accuracy: Benefits of measuring response time in computerised testing. Australasian Journal of Educational Technology.

[CR24] Hambleton RK, Swaminathan H, Rogers HJ (1991). *Fundamentals of item response theory*.

[CR25] Hart S, Staveland L (1988). Development of NASA-TLX (task load index): Results of empirical and theoretical research. Advances in Psychology.

[CR26] Johannsen G, Moray N (1979). Workload and workload measurement. *Mental workload: Its theory and measurement,* (pp. 3–11).

[CR27] Jung J (2018). Effects of task complexity and working memory capacity on L2 reading comprehension. System.

[CR28] Karami H (2010). *A differential item functioning analysis of a language proficiency test: An investigation of background knowledge bias* [Unpublished MA Thesis].

[CR29] Krell M, Khan S, van Driel J (2021). Analyzing Cognitive Demands of a Scientific Reasoning Test Using the Linear Logistic Test Model (LLTM). Education Sciences.

[CR30] Lee H (2014). Measuring cognitive load with electroencephalography and self-report: Focus on the effect of English-medium learning for Korean students. Educational Psychology.

[CR31] Lee J (2019). Task complexity, cognitive load, and L1 speech. Applied Linguistics.

[CR32] Leppink J (2017). Cognitive load theory: Practical implications and an important challenge. Journal of Taibah University Medical Sciences.

[CR33] Linacre JM (2013). Winsteps® (Version 3.80.1) [Computer Software].

[CR34] Marcus N, Cooper M, Sweller J (1996). Understanding instructions. Journal of Educational Psychology.

[CR35] Martin S (2014). Measuring cognitive load and cognition: Metrics for technology enhanced learning. Educational Research and Evaluation: An International Journal on Theory and Practice.

[CR36] Minkley N, Xu KM, Krell M (2021). Analyzing relationships between causal and assessment factors of cognitive load: associations between objective and subjective measures of cognitive load, stress, interest, and self-concept. Frontiers in Education.

[CR37] Moreno R (2010). Cognitive load theory: More food for thought. Instructional Science.

[CR38] O’Donnell RD, Eggemeier FT, Boff KR, Kaufman L, Thomas JP (1986). Workload assessment methodology. *Handbook of perception and human performance, Vol. 2. Cognitive processes and performance*.

[CR39] Paas F (1992). Training strategies for attaining transfer of problem-solving skill in statistics: A cognitive-load approach. Journal of Educational Psychology.

[CR40] Paas F, Renkl A, Sweller J (2003). Cognitive load theory and instructional design: Recent developments. Educational Psychologist.

[CR41] Paas F, Tuovinen JE, Tabbers H, van Gerven PWM (2003). Cognitive load measurements as a means to advance cognitive load theory. Educational Psychologist.

[CR42] Paas F, van Merriënboer JJG (1993). The efficiency of instructional conditions: An approach to combine mental effort and performance measures. Human Factors.

[CR43] Pallant J (2016). *SPSS survival manual: A step by step guide to data analysis using IBM SPSS*.

[CR44] Peirce J, Gray JR, Simpson S, MacAskill M, Höchenberger R, Sogo H, Kastman E, Lindeløv JK (2019). Psychopy2: Experiments in behavior made easy. Behavior Research Methods.

[CR45] Pelánek R, Effenberger T, Čechák J (2022). Complexity and difficulty of items in learning systems. International Journal of Artificial Intelligence in Education.

[CR46] Ponce HR, Mayer RE, Sitthiworachart J, Lopez MJ (2020). Effects on response time and accuracy of technology-enhanced cloze tests: An eye-tracking study. Educational Technology Research and Development.

[CR47] Pouw WT, Eielts C, van Gog T, Zwaan RA, Paas F (2016). Does (non-)meaningful sensori-motor engagement promote learning with animated physical systems?. Mind, Brain, and Education.

[CR48] Prisacari AA, Danielson J (2017). Computer-based versus paper-based testing: Investigating testing mode with cognitive load and scratch paper use. Computers in Human Behavior.

[CR49] Rasch G (1960). *Probabilistic models for some intelligence and attainment tests*.

[CR50] Révész A, Michel M, Gilabert R (2016). Measuring cognitive task demands using dual-task methodology, subjective self-ratings, and expert judgments: A validation study. Studies in Second Language Acquisition.

[CR51] Révész A, Sachs R, Hama M (2014). The effects of task complexity and input frequency on the acquisition of the past counterfactual construction through recasts. Language Learning.

[CR52] Robinson P (2001). Task complexity, task difficulty, and task production: Exploring interactions in a componential framework. Applied Linguistics.

[CR53] Sasayama S (2016). Is a ‘complex’ task really complex? Validating the assumption of cognitive task complexity. The Modern Language Journal.

[CR54] Scheiter K, Ackerman R, Hoogerheide V (2020). Looking at mental effort appraisals through a metacognitive lens: Are they biased?. Educational Psychology Review.

[CR55] Shohamy E, Donitsa-Schmidt S, Ferman I (1996). Test impact revisited washback effect over time. Language Testing.

[CR56] Skulmowski A, Rey GD (2017). Measuring cognitive load in embodied learning settings. Frontiers in Psychology.

[CR57] Sweller J (1988). Cognitive load during problem solving: Effects on learning. Cognitive Science.

[CR58] Sweller J (1994). Cognitive load theory, learning difficulty, and instructional design. Learning and Instruction.

[CR59] Sweller J, Elen J, Clark RE (2006). How the human cognitive system deals with complexity. *Handling complexity in learning environments*.

[CR60] Sweller J, Plass JL, Moreno R, Brünken R (2010). *Cognitive load theory: Recent theoretical advances*. *Cognitive load theory*.

[CR61] Sweller J (2020). Cognitive load theory and educational technology. Educational Technology Research and Development.

[CR62] Sweller J, Ayres P, Kalyuga S (2011). *Cognitive load theory*.

[CR63] Sweller J, van Merriënboer JJG, Paas F (1998). Cognitive architecture and instructional design. Educational Psychology Review.

[CR64] Sweller J, van Merriënboer JJG, Paas F (2019). Cognitive architecture and instructional design: 20 years later. Educational Psychology Review.

[CR65] van der Linden WJ (2009). Conceptual issues in response-time modeling. Journal of Educational Measurement.

[CR66] van Gog T, Paas F (2008). Instructional efficiency: Revisiting the original construct in educational research. Educational Psychologist.

[CR67] Young MS, Brookhuis KA, Wickens CD, Hancock PA (2015). State of science: Mental workload in ergonomics. Ergonomics.

